# The additional value of lung cancer screening program in identifying unrecognized diseases

**DOI:** 10.1186/s12890-022-01826-1

**Published:** 2022-01-31

**Authors:** Panaiotis Finamore, Luigi Tanese, Filippo Longo, Domenico De Stefano, Claudio Pedone, Laura Angelici, Nera Agabiti, Silvia Cascini, Marina Davoli, Bruno Beomonte Zobel, Raffaele Antonelli Incalzi, Pierfilippo Crucitti

**Affiliations:** 1 Unit of Geriatrics, Department of Medicine and Surgery, Campus Bio-Medico University and Teaching Hospital, Rome, Italy; 2 Unit of Imaging Center, Department of Medicine and Surgery, Campus Bio-Medico University and Teaching Hospital, Rome, Italy; 3 Unit of Thoracic Surgery, Department of Medicine and Surgery, Campus Bio-Medico University and Teaching Hospital, Rome, Italy; 4 Dipartimento di Epidemiologia del Servizio Sanitario Regionale, Regione Lazio, ASL Roma 1, Rome, Italy

**Keywords:** Computed tomography, Lung cancer, Screening, Cardiovascular disease, Respiratory disorders

## Abstract

**Background:**

A systematic examination of low-dose CT (LDCT) scan, beside lung nodules, may disclose the presence of undiagnosed diseases, improving the efficacy and the cost/efficacy of these programs. The study was aimed at evaluating the association between LDCT scan findings and non-oncologic and oncologic diseases.

**Methods:**

The LDCT scan of participants to the “Un Respiro per la vita”^®^ lung cancer screening program were checked and abnormal findings, beside lung nodules, recorded. First admission to the acute care because of cardiovascular (CD), respiratory (RD) and oncological diseases (OD) in the following three years were retrieved. The association of LDCT scan abnormal findings with CD, RD and OD was assessed through univariable and multivariable logistic regression models.

**Results:**

Mean age of 746 participants was 62 years (SD:5), 62% were male. 11 (1.5%) received a diagnosis of lung cancer. 16.1% participants were admitted to the acute care in the following three years: 8.6% for CD, 4.3% for RD and 5.2% for OD. Valve calcification (OR 2.02, p:0.02) and mucus plugs (OR 3.37, p:0.04) were positively associated with CD, while sub-pleural fibrosis had a protective role (OR 0.47, p:0.01). Lung nodules > 8 mm (OR 5.54, p: < 0.01), tracheal deviation (OR 6.04, p:0.01) and mucus plugs (OR 4.00, p:0.04) were positively associated with OD admissions. Centrilobular emphysema OR for RD admissions was 1.97 (p:0.06).

**Conclusions:**

The observed association between selected LDCT findings and ensuing CD, RD and OD suggests that the information potential of LCDT goes beyond the screening of lung cancer.

**Supplementary Information:**

The online version contains supplementary material available at 10.1186/s12890-022-01826-1.

## Background

Annual screening for lung cancer in high risk populations through low dose computed tomography (LDCT) has gained popularity and is currently recommended by US Preventive Services Task Force (USPSTF) after the National Lung Cancer Screening Trial (NLCST) could demonstrate a 20% decrease in lung cancer mortality in people aged 55–80 years with a smoke history of at least 30 pack year, either current smokers or quitters from within 15 years [1, 2]. Two smaller subsequent trials, the Danish Lung Cancer Screening Trial (DLCST) and the Detection and Screening of Early Lung Cancer by Novel Imaging Technology and Molecular Essays (DANTE), failed to confirm these favorable results [3, 4]. Indeed, in both trials the annual LDCT screening did not provide any survival benefit. Differences in the studied populations might partly account for discrepancy as a lower risk population was enrolled and a lower incidence of lung cancer was observed in both DLCST and DANTE trials. Selected differences in the definition of the suspicious lung nodule and in the follow up strategy are a further source of heterogeneity among the three studies. Interestingly, in all mentioned studies the low specificity of screening raised concern about the risk related to undue exposure to radiation and other unnecessary diagnostic procedures [2–4]. Also in the UKLS trial 1994 people had to be screened and then, to be exposed to radiation for 42 subjects being diagnosed with lung cancer. Secondary analyses of these trials showed that restricting the screening to higher risk people might be a convenient strategy to improve specificity [5]. Furthermore, an algorithm has been proposed to identify people most likely to benefit from lung cancer screening [6, 7]. Finally, in 2016 Katki et al. proposed a risk computation model and validated it in both the Prostate, Lung, Colorectal, and Ovarian Cancer Screening Trial (PLCO; 1993–2009) and the NLCST [8]. Such a model could avert a further 20% more deaths with regard to NLCST and spare an important proportion of unnecessary screenings. On the other hand, both the MILD trial and a meta-analysis of 4 trials failed to show any 5 year survival benefit of the screening procedure [9].

Data obtained through screening may be improved and refined by secondary analysis. For instance, the NELSON study convincingly demonstrated that new onset pulmonary nodules, i.e. nodules which were absent at baseline, carry a dismal prognosis and, as such, should be followed more aggressively [10]. Nevertheless, this useful information does not impact the basic limitation of any LDCT based screening procedure, the low specificity. Thus, the development of molecular biomarkers is eagerly awaited to improve the selection procedure, but to date the available biomarkers do not meet the required quality standards [11].

While the controversy about cost/efficacy of lung cancer screening will probably end with the joined elaboration of NELSON and UKLS data, we hypothesize that a more comprehensive and not exclusively oncologic approach to results from LDCT might disclose the full value of the screening. Indeed, there are many important non oncological conditions which might be disclosed by LDCT. Among those are lung fibrosis and bronchiectasis. Furthermore, non-pulmonary findings such as coronary artery calcium and vertebral density and structure might be a further valuable information from LDCT. Finally, selected LDCT features might herald lung cancer in subjects presently free from it. On these bases, as a proof of concept we planned to re-analyze comprehensively the first 1000 LDCT scans of people enrolled from April 2014 to May 2015 in the lung cancer screening program hosted in our Institution with the aim of catching the added non oncologic and oncologic value of the screening. Furthermore, we purposed to verify whether LDCT findings have prognostic and not only diagnostic potential towards cancer.

## Materials and methods

### Study design and sources of data

This is a secondary analysis of an ongoing mono-center and observational prospective study carried out at Campus Bio-Medico University and Teaching Hospital (CBM) in Rome, Italy. In this proof of concept study were consecutively included the first 1000 participants who performed a LDCT scan from April 2014 to May 2015, and their admission to the acute care in the following three years for cardiovascular, respiratory and oncological disease (CD, RD, OD) was investigated. Standardized record linkage procedures using a unique code identifier were applied to link clinical datasets with the regional population registry (PR) that comprised the place of residence and dates of health insurance coverage for all enrollees of the Lazio Regional Health Service. Death certificates from the regional mortality register were used to update the PR. All data sources can be linked between them using anonymous key according to the national privacy law (national legislative decree on privacy policy n. 196/30 June 2003). The Regional Health Information System databases (ICD-9-CM codes of the International Classification of Diseases) was used to identify the outcomes. The Department of Epidemiology of Lazio Regional Health Service is the regional referral center for epidemiological research and has full access to anonymized health information systems. This study conforms to the principles outlined in the Declaration of Helsinki and it was approved by the Ethical Commission of the Campus Bio-Medico University (protocol:34/15 PAR ComEt CBM). All the study participants provided written informed consent.

### Setting

“Un Respiro per la Vita”^®^ (Breath for Life) is a lung cancer screening program funded by “Fondazione Un Respiro per la Vita Onlus^®^” which offers, free of charge, the opportunity to perform a LDCT scan to eligible candidates, i.e. people aged 55–75 years deemed at risk for developing lung cancer given their smoking history (current smokers or quitters from within10 years), exposure to carcinogens (e.g. asbestos, radiation, etc.) or history of chronic obstructive pulmonary disease (COPD). Exclusion criteria are: current oxygen therapy and history of cancer, apart from localized prostatic cancer, in situ uterine cervix cancer and non-melanoma skin cancer, in the previous five years. Candidates underwent a preliminary outpatient visit where a multidisciplinary team evaluates eligibility criteria and collects socio-demographic information. Then, participants performed a LDCT scan. LDCT was preferred to CT scan to reduce radiation-induced damages, as recommended by the European Respiratory Society and the American Thoracic Society [12, 13]. A description of the management of positive findings, defined as nodule equal or larger than 5 mm, is out of the aim of the present study and can be found elsewhere [14, 15]. For the purposes of the present study we will limit the analysis to people enrolled according to standard criteria [2, 16], but data pertaining to the whole enrolled population will also be provided.

### Low dose CT-scan

LDCT scans were performed from the lung apices to bases in a single breath hold at 120 kVp, 30 mA, 1.4:1 pitch ratio. The axial images were collimated at 0.6 mm and reconstructed at 3 mm slice thickness, using both the bone and soft tissue algorithms. No intravenous contrast material was administrated. All CT examinations were performed using a 64-detector row CT scanner (Siemens Somatom Sensation, Erlangen). Two radiologists involved in the study, with 5 years of experience in chest CT scan interpretation, were instructed to report respiratory findings (lung, bronchial tree or pleural alterations), beside the identification of lung nodules, cardiovascular findings and other findings (e.g. breast nodules, hiatal hernia). A detailed list of LDCT considered findings can be found in Additional file 1 (see *e-Table **1*). Where was impossible to perform a quantitative evaluation to establish the effective presence of a radiological finding, a quality assessment was already made. The radiologist readers coded such findings as “present” (score 1) or “absent” (score 0) in a double-blind mode.

**Table 1:** Participants’ characteristics and LDCT scan findings (n = 746)

Variables	N = 746
**Age (years)**	
52–58	203 (27.2)
59–64	306 (41.0)
65–78	237 (31.8)
**Sex (M)**	462 (61.9)
**Smokers**	540 (72.4)
**Ex-smokers**	206 (27.8)
**Pack year**	49.5 (19.9)
**Professional exposure**	2 (0.27)
**Cancer pathology familiarity**	359 (48.1)
**Comorbidities**	
Asthma	12 (1.6)
Diabetes mellitus	57 (7.6)
Hypertension	260 (34.9)
Gastroesophageal reflux disease	34 (4.6)
Hypercholesterolemia	136 (18.2)
Hyperthyroidism	1 (0.1)
Benign prostatic hyperplasia (BPH)	47 (6.3)
**Lung nodules**	
Nodules < = 4 mm	174 (23.3)
Nodules 5–6 mm	135 (18.1)
Nodules 7–8 mm	43 (5.8)
Nodules > = 8 mm	98 (13.1)
**LDCT findings**	
Ground glass opacity	47 (6.3)
Centrilobular emphysema	227 (30.4)
Paraseptal emphysema	82 (11)
Atelectasis areas	68 (9.1)
Bronchial wall thickening	291 (39)
Bronchiectasis	62 (8.3)
Diffuse fibrosis	14 (1.9)
Subpleural fibrosis	277 (37.1)
Paravertebral fibrosis	25 (3.4)
Mucus plugs	17 (2.3)
Tracheal deviation	18 (2.4)
Focal pleural thickening	58 (7.8)
Pleural effusion	0 (0)
Vascular Calcifications	356 (47.7)
Coronary Calcifications	276 (37)
Valve Calcifications	119 (16)
Breast nodule	12 (1.6)

Categorical variables are expressed as frequency (%), while continuous variables as mean (SD)

### Outcome assessment

The incidence of the first event of an acute hospitalization was retrieved from the Regional HIS. Hospital electronic records of participants up to three years from the enrollment were reviewed to ascertain the incidence of acute admission for cardiovascular (CD), respiratory (RD) or oncological disease (OD). First hospital CD admission was defined on the basis of main diagnosed ICD9CM codes: “Cardiovascular Disease” (codes 390–409, 415–425 and 439–459); “Coronary Heart Disease, Ischemic Cardiomyopathy” (codes 410–414, 429.7); “Arrhythmias” (codes 426, 427), “Hypertension” (codes 401–405), “Heart failure” (code 428) and “Cerebrovascular Disease” (codes 430, 432–438). First hospital RD admission was defined as on the basis of main diagnosis ICD9CM codes 460–519, involving acute and chronic respiratory diseases (e.g. pneumonia, COPD). Finally, first hospital OD admission was defined on the basis of main diagnosis ICD9CM codes 140-208.9 and V10, thus not limited to lung cancer.

### Statistical analysis

Data were reported using mean and standard deviations (SD) or median and interquartile range (IQR) for continuous variables, and number and percentage (%) for categorical variables. In order to make our results comparable with those of previous lung cancer screening trials, we focused our main analyses to the individuals who performed LDCT scans because of their smoking history (n = 746), excluding those eligible by exposure to carcinogens and by history of COPD (n = 228). However, results of the analyses performed on the whole screened sample with available follow up are reported in supplemental material (see Additional file 1: e-Table S2, e-Table S3, e-Table S4 and e-Table S5). The inter-rater reliability of radiologists was assessed using the Cohen’s kappa. The association of LDCT scan findings (*exposure variables*) and the incidence of CD, RD and OD acute hospitalization (*outcomes*) was assessed using logistic regression in a crude model and after adjusting for age, sex and pack year, considered as potential confounders. All the analyses were performed using the Statistical Analysis System (SAS) statistical software package, version 9.4. SAS Institute Inc., Cary, NC, USA.

## Results

### Participants

**Fig. 1:**
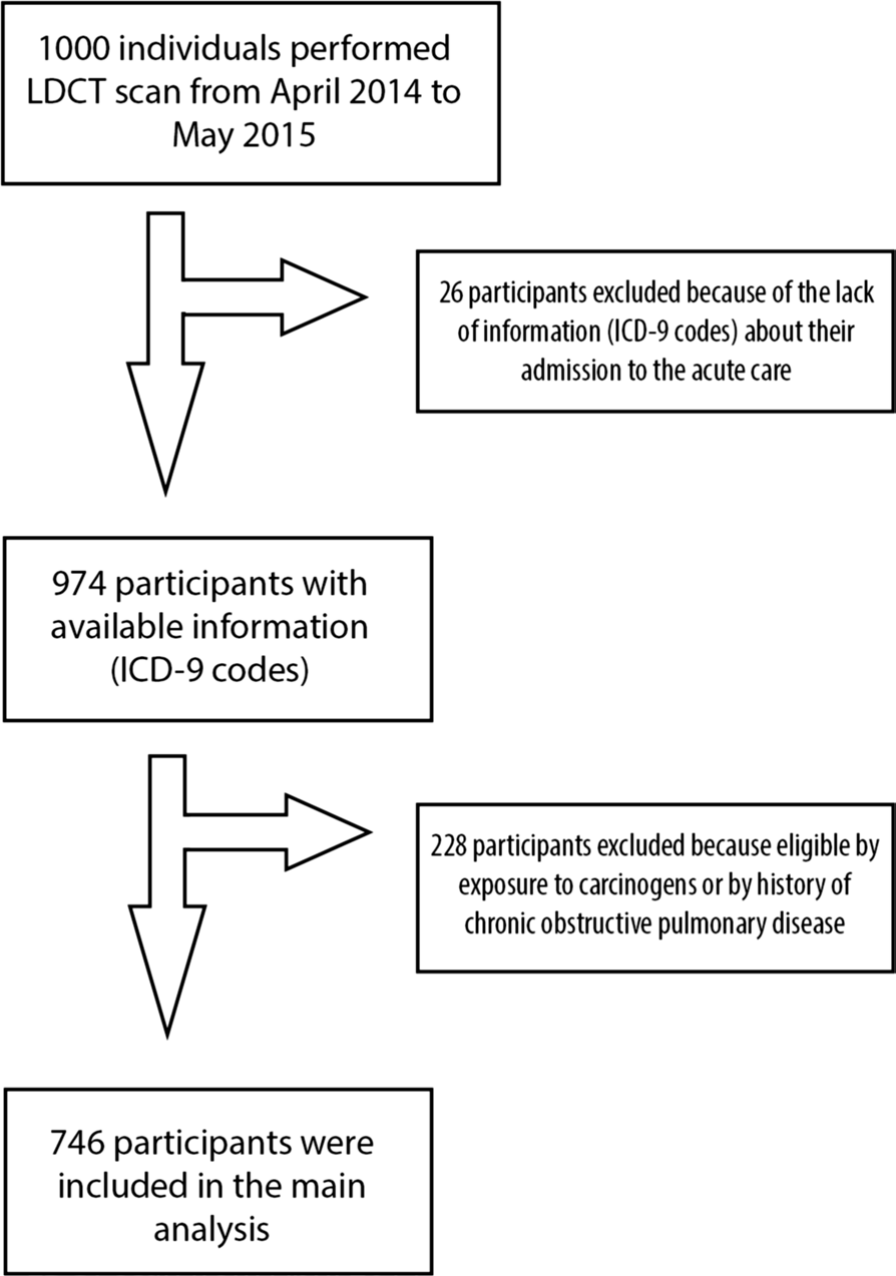
Flow chart of the enrollment

A total of 1000 individuals entered the lung cancer screening program between April 2014 to May 2015: 974 (97.6%) had available data in the Lazio Region PR. The 74.7% (n = 746) was eligible because of the smoking history according to criteria shared by the vast majority of such trials and, thus, was the primary object of our study (see Fig. 1). The remaining 25.3% (n = 228) was enrolled due to professional exposure or a history of COPD. Participants had a mean age of 62 years (SD:5), 62% were male and 72% were current smokers; mean pack year was 49.5 (SD: 19.9). Table 1 summarize participants’ characteristics and LDCT findings.

### Inter rater agreement

The kappa Cohen’s coefficient disclosed a good to high concordance between raters (kappa ≥ 0.50, p < 0.001) for the vast majority of the LDCT scan findings of interest. The only findings for which the agreement did not reach this threshold were: mucus plugs and thickening of bronchial tree. A detailed list of kappa coefficients for individual findings of interest is reported in Additional file 1: e-Table S6.

### Lung cancer screening

276 (37%) participants had a positive finding at LDCT scan, 174 (23.3%) had nodule/s lower than 5 mm and 296 (39.7%) were completely free from nodules. 14 (1.9%) screened individuals were addressed to biopsy. 11 (1.5%) were positive for primitive lung cancer: 10 (91%) were adenocarcinoma and 1 (9%) was squamous cell lung cancer. Of the 7 patients with lung cancer who underwent surgery in our Institution, 4 were in stage Ia-Ib.

### Occurrence of CD, RD and OD admissions

During the follow-up, 120 (16.1%) participants were admitted to the acute care: 64 (8.6%) for CD, 32 (4.3%) for RD and 39 (5.2%) for OD. Among OD admissions, 16 (41%) were for lung cancer and 23 (59%) for other oncologic diseases, mainly breast cancer (6 participants) and genitourinary cancer (5 participants). Of the 16 patients admitted for lung cancer, 11 were those already diagnosed at baseline and 5 were new diagnoses in patients addressed to follow-up for their lung nodules. The median time to the occurrence was 479 days for CD, 467 for RD and 353 for OD.

### Association of LDCT scan findings and outcomes

Mucus plugs (OR 3.37, 95%CI 1.04–10.89, p 0.04) and valve (OR 2.02, 95%CI 1.11–3.67, p 0.02), but not coronary (OR 1.56, 95%CI 0.91–2.66, p 0.10), calcifications were positively associated with CD admissions in the adjusted model, while subpleural fibrosis showed a negative association (OR 0.47, 95%CI 0.26–0.86, p 0.01) (see Table 2).

**Table 2:** Association between participants’ characteristics and LDCT scan findings with cardiovascular diseases (n = 64)

		**Univariable logistic regression**			**Multivariable logistic regression**		
	**N = 64**	**OR**	**95%CI**	**P**	**OR**	**95%CI**	**P**
**Age**							
52–58 years	10 (15.6)	Ref			–	–	–
59–64 years	30 (46.9)	2.10	1.00–4.39	0.05	–	–	–
65–78 years	24 (37.5)	2.18	1.01–4.66	0.05	–	–	–
**Sex (M)**	49 (76.6)	2.13	1.17–3.87	0.01	–	–	–
**Smoking**							
Former smoker	11 (17.2)	Ref			–	–	–
Current smoker	53 (82.8)	1.93	0.99–3.77	0.05	–	–	–
Pack year	49 (19.7)	1.01	1.00–1.02	0.05	–	–	–
**Lung nodules**							
No nodules	24 (37.5)	Ref			Ref		
Nodules < = 8 mm	33 (51.6)	1.13	0.64–2.00	0.68	1.11	0.62–1.97	0.73
Nodules > 8 mm	7 (10.9)	1.05	0.51–2.17	0.89	0.96	0.46–1.99	0.46
**Ground glass opacity**	4 (6.3)	0.99	0.34–2.85	0.99	1.10	0.38–3.21	0.38
**Centrolobular emphysema**	20 (31.3)	1.04	0.60–1.81	0.88	0.96	0.55–1.68	0.87
**Paraseptal emphysema**	9 (14.1)	1.37	0.65–2.88	0.41	1.22	0.57–2.59	0.61
**Atelectasis areas**	6 (9.4)	1.04	0.43–2.50	0.94	1.01	0.41–2.45	0.99
**Bronchial wall thickening**	30 (46.9)	1.42	0.85–2.38	0.18	1.33	0.79–2.24	0.28
**Bronchiectasis**	6 (9.4)	1.16	0.48–2.80	0.75	1.14	0.47–2.79	0.77
**Diffuse fibrosis**	3 (4.7)	3.00	0.82–11.04	0.10	2.49	0.65–9.51	0.18
**Subpleural fibrosis**	15 (23.4)	0.49	0.27–0.89	0.02	0.47	0.26–0.86	0.01
**Paravertebral fibrosis**	4 (6.3)	2.10	0.70–6.31	0.19	1.90	0.62–5.83	0.26
**Mucus plugs**	4 (6.3)	3.43	1.08–10.83	0.04	3.37	1.04–10.89	0.04
**Tracheal deviation**	1 (1.6)	0.63	0.08–4.84	0.66	0.45	0.06–3.55	0.45
**Coronary calcifications**	33 (51.6)	1.92	1.15–3.22	0.01	1.56	0.91–2.66	0.10
**Valve calcifications**	18 (28.1)	2.25	1.26–4.04	< 0.01	2.02	1.11–3.67	0.02
**Breast nodule**	2 (3.1)	2.17	0.47–10.12	0.32	3.33	0.67–16.66	0.14

Categorical variables are expressed as frequency (%), while continuous variables as mean (SD). Adjustment variables in the multivariable logistic regression were age, sex and pack year. Broncholitiasis was not represented in this cohort

Emphysema, mainly when centrilobular, was associated with RD admissions in the univariable, but not in the adjusted model (OR 1.97, 95%CI 0.96–4.05, p 0.06) (see Table 3). However, when considering a composite outcome of neoplastic and non-neoplastic respiratory disease centrilobular emphysema showed a positive association (OR 2.00, 95%CI 1.10–3.63, p 0.02).

**Table 3:** Association between participants’ characteristics and LDCT scan findings with respiratory diseases (n = 32)

		**Univariable logistic regression**			**Multivariable logistic regression**		
	**N = 32**	**OR**	**95%CI**	**P**	**OR**	**95%CI**	**P**
**Age**							
52–58 years	7 (21.9)	Ref			–	–	–
59–64 years	13 (40.6)	1.24	0.49–3.17	0.65	–	–	–
65–78 years	12 (37.5)	1.49	0.58–3.87	0.41	–	–	–
**Sex (M)**	25 (78.1)	2.26	0.97–5.30	0.06	–	–	–
**Smoking**							
Former smoker	8 (25)	Ref			–	–	–
Current smoker	24 (75)	1.15	0.51–2.61	0.73	–	–	–
Pack year	49.2 (19.7)	1.01	1.00–1.03	0.05	–	–	–
**Lung nodules**							
No nodules	12 (37.5)	Ref			Ref		
Nodules < = 8 mm	11 (34.4)	0.76	0.33–1.76	0.53	0.77	0.33–1.78	0.54
Nodules > 8 mm	9 (28.1)	2.39	0.98–5.87	0.06	2.11	0.85–5.24	0.11
**Ground glass opacity**	1 (3.13)	0.47	0.06–3.51	0.46	0.51	0.07–3.83	0.51
**Centrilobular emphysema**	15 (46.9)	2.09	1.03–4.26	0.04	1.97	0.96–4.05	0.06
**Paraseptal emphysema**	7 (21.9)	2.39	1.00–5.70	0.05	2.10	0.87–5.07	0.10
**Atelectasis areas**	3 (9.4)	1.03	0.31–3.48	0.96	1.02	0.30–3.48	0.97
**Bronchial wall thickening**	11 (34.4)	0.81	0.39–1.71	0.96	0.75	0.35–1.59	0.45
**Bronchiectasis**	1 (3.1)	0.35	0.05–2.57	0.30	0.34	0.05–2.56	0.29
**Diffuse fibrosis**	2 (6.3)	3.90	0.84–18.21	0.08	3.16	0.65–15.51	0.16
**Subpleural fibrosis**	11 (34.4)	0.88	0.42–1.86	0.74	0.86	0.40–1.82	0.69
**Paravertebral fibrosis**	1 (3.1)	1.04	0.12–7.08	0.94	0.81	0.11–6.31	0.84
**Mucus plugs**	1 (3.1)	1.41	0.18–10.94	0.74	1.40	0.18–11.03	0.75
**Coronary calcifications**	16 (50)	1.75	0.86–3.55	0.12	1.45	0.69–3.03	0.33
**Valve calcifications**	5 (15.6)	0.98	0.37–2.58	0.96	0.86	0.32–2.32	0.76
**Breast nodule**	1 (3.1)	2.06	0.26–16.48	0.49	3.54	0.40	0.25

Categorical variables are expressed as frequency (%), while continuous variables as mean (SD). Adjustment variables in the multivariable logistic regression were age, sex and pack year. The following LDCT scan findings were not represented in this cohort: bronchiolitis, bronchiolitiasis, tracheal deviation, pulmonary trunk ectasia, pericardium pouring, thoracic cage abnormalities and hiatal hernia

Lung nodules > 8 mm (OR 5.54, 95%CI 2.45–12.54, p < 0.01), tracheal deviation (OR 6.04, 95%CI 1.49–24.4, p 0.01) and mucus plugs (OR 4.00, 95%CI 1.05–15.18, p 0.04) showed a positive associations with OD admissions (see Table 4).

**Table 4:** Association between participants’ characteristics and LDCT scan findings with oncologic diseases (n = 39)

		**Univariable logistic regression**			**Multivariable logistic regression**		
	**N = 39**	**OR**	**95%CI**	**P**	**OR**	**95%CI**	**P**
**Age**							
52–58 years	7 (18)	Ref			–	–	–
59–64 years	14 (35.9)	1.34	0.53–3.38	0.53	–	–	–
65–78 years	18 (46.1)	2.30	0.94–5.62	0.07	–	–	–
**Sex (M)**	18 (46.1)	0.51	0.26–0.97	0.04	–	–	–
**Smoking**							
Former smoker	7 (18)	Ref			–	–	–
Current smoker	32 (82)	1.79	0.78–4.12	0.17	–	–	–
Pack year	52.4 (27.6)	1.01	0.99–1.02	0.33	–	–	–
**Lung nodules**							
No nodules	11 (28.2)	Ref			Ref		
Nodules < = 8 mm	11 (28.2)	0.84	0.36–1.96	0.68	0.77	0.33–1.82	0.55
Nodules > 8 mm	17 (43.6)	5.44	2.45–12	< 0.01	5.54	2.45–12.54	< 0.01
**Ground glass opacity**	3 (7.7)	1.25	0.37–4.24	0.71	1.12	0.33–3.83	0.86
**Centrilobular emphysema**	14 (35.9)	1.30	0.66–2.55	0.45	1.34	0.67–2.67	0.40
**Paraseptal emphysema**	6 (15.4)	1.51	0.61–3.72	0.37	1.49	0.59–3.73	0.40
**Atelectasis areas**	6 (15.4)	1.89	0.76–4.69	0.17	1.92	0.76–4.84	0.17
**Bronchial wall thickening**	18 (46.1)	1.36	0.71–2.60	0.35	1.25	0.64–2.42	0.51
**Bronchiectasis**	3 (7.7)	0.91	0.27–3.06	0.88	0.78	0.23–2.65	0.69
**Subpleural fibrosis**	14 (35.9)	0.94	0.48–1.85	0.87	0.88	0.45–1.73	0.71
**Paravertebral fibrosis**	2 (5.1)	1.61	0.36–7.08	0.53	1.56	0.35–6.95	0.56
**Mucus plugs**	3 (7.7)	4.12	1.13–14.99	0.03	4	1.05–15.18	0.04
**Tracheal deviation**	3 (7.7)	4.58	1.23–17	0.02	6.04	1.49–24.4	0.01
**Coronary calcifications**	17 (43.6)	1.34	0.7–2.56	0.38	1.39	0.70–2.76	0.35
**Valve calcifications**	8 (20.5)	1.38	0.62–3.09	0.43	1.21	0.53–2.77	0.64
**Breast nodule**	1 (2.6)	1.66	0.21–13.23	0.63	1.04	0.12–8.58	0.12

Categorical variables are expressed as frequency (%), while continuous variables as mean (SD). Adjustment variables in the multivariable logistic regression were age, sex and pack year. The following LDCT scan findings were not represented in this cohort: diffuse fibrosis, pulmonary trunk ectasia, pericardium pouring and thoracic cage abnormalities

With the exception of the positive association of RD admissions with centrilobular (OR 2.12, 95%CI 1.13–3.97, p 0.02) and paraseptal emphysema (OR 2.69, 95%CI 1.26–5.74, p 0.01), the associations between LDCT scan findings and outcomes did not change with the inclusion of the 228 individuals who entered the screening program because of the exposure to carcinogens or as affected by COPD (see Additional file 1: e-Table S 2, e-Table S 3, e-Table S 4 and e-Table S 5).

A positive association was found between age, male sex (OR 2.13, 95%CI 1.17–3.87, p 0.01), smoking habit (smoker vs quitter OR 1.93, 95%CI 0.99–3.77, p 0.05) and cumulative smoking exposure (pack year: OR 1.01, 95%CI 1.00–1.02, p 0.05) with CD admissions, although the last two associations were not statistically significant. No association was instead found between age, sex and smoking habit with RD admissions, albeit a positive association, even not statistically significant, was found between pack year and RD admissions (OR 1.01, 95%CI 1.00–1.03, p 0.05). Furthermore, age and smoking habit and cumulative exposure did not show any association with OD admissions, while a negative and significant association was found between male sex (OR 0.51, 95%CI 0.26–0.97, p 0.04) and OD admissions.

## Discussion

Our analysis shows that selected LDCT scan findings in variable proportion qualify as harbingers of cardiovascular, respiratory and oncologic diseases, as identified on the basis of related acute care admissions in the three years following the LDCT scan screening. Thus, besides identifying eleven subjects amenable to surgery for early stage lung cancer, LDCT scan provided an additional information which seems worthy of being carefully assessed.

Mucous plugs were the main predictors of cardiovascular events, despite being very rare. Indeed, 4 out of the 17 subjects having mucous plugs had a cardiovascular event. Thus, the fact that this pulmonary finding reflecting inflammation predicted a cardiovascular outcome suggests that systemic inflammation as a spill over of pulmonary inflammation promotes atherosclerosis and related diseases. This is on line with a bulk of basic [17, 18] and clinical [19] research findings, and with the evidence in the whole screened sample that bronchiectasis has a positive association with CD (see e-Table 3). Interestingly, valve calcifications, but not vascular calcifications, were cardiovascular findings associated with a cardiovascular outcome. Valve calcifications are commonly expression of valve degeneration in older population [20]. Once considered to be the result of a passive process, nowadays they are ascribed to endothelial dysfunction and inflammation promoting the extracellular matrix remodelling, and eventually the bone deposition leading to valve calcification [21]. This interpretation strengthens the hypothesis that inflammation underpins the association between valve calcification and CD occurrence. Furthermore, the negative association between subpleural fibrosis and CD is rather than intuitive. Sub-pleural fibrosis likely represents the effect of pneumonia, metapneumonic fibrosis. Since patients who experience an important pneumonia likely are more motivated to quit smoking, this seems the most likely explanation of the protective role of subpleural fibrosis.

Emphysema was associated with respiratory non neoplastic events, mainly pneumonia [n = 8 (1.1%)] and exacerbated COPD [n = 22 (2.3%)]. Structural abnormalities of the lung are expected to correlate mainly with manifestations of chronic respiratory conditions like COPD rather than with pneumonia, however smoke exposure and lung damage are also related to impaired organ immunity and, then, to the risk of respiratory infections [22, 23]. Furthermore, mucus plugs were not associated with RD. Since hospital admissions are the largest determinant of the direct medical costs related to COPD exacerbations [24], only severe cases are hospitalized, and the same is for pneumonia, therefore the lack of association might at least in part depend on the underestimation of the RD occurrence.

The detection rate of the screening program was 0.011%, and raised to 0.016% considering the 5 participants who received the diagnosis of lung cancer during the follow-up. However, considering the identification of other types of cancer (e.g. breast cancer) among participants addressed to follow-up for their nodules, the detection rate of this screening is even higher, and may further increase including individuals with an exposure to carcinogens and with COPD who are more likely to develop cancer. Associated with an incident OD were nodules exceeding 8 mm, which is not surprising, and also tracheal deviation and mucus plugs. Tracheal deviation usually reflects advanced lung cancer, but this is not the case since the maximum diameter of the nodules was ≤ 4 cm. Thus, it might be a chance finding or reflect the occurrence of tracheal deviations as an effect of asymmetrical emphysema or fibrosis, which are well known risk factors for cancer. Indeed, only 5% of tracheal deviations were not associated with a shift of the mediastinum due to emphysema, bronchiectasis or other chronic lung abnormalities. Finally, mucus plugs might represent the epiphenomenon of chronic lung inflammation, which represent a widely recognized risk factor for lung and non-lung cancers [25, 26], or be the expression of advanced lung disease, which likely explains their association with OD.

### Strength and limitations

Limitations of this study are many. First, the outcome was a proxy outcome; likely it does not cover the full spectrum of events. However, the well standardized computation method guarantees for these data defining a true, albeit somehow incomplete, outcome. Second, the concordance between readers varied across the different LDCT scan findings, ranging from 0.26 (95%CI 0.15–0.67) for mucus plugs to 0.92 (95%CI 0.77–1.07) for paraseptal emphysema (see e-Table 6). This variability, though in a range consistent with fine to good agreement for almost all the considered LDCT scan findings, might partly affect the strength of the associations. However, it seemed logical to discuss them because a proof of concept study like this requires a liberal approach while waiting for a larger validation study. Finally, the available information prevented us from testing selected hypotheses to explain the observed correlations.

## Conclusion

Though preliminary and obtained on a small population, these data highlight the potential of LDCT based lung cancer screening for identifying patients at greater risk of CD and RD besides OD. They show that selected LDCT findings have multiple prognostic properties in a population with smoking exposure over the threshold for screening eligibility, and, thus, are worthy of being carefully searched for and recorded. However, targeting people at risk of CD, RD or OD does not automatically mean that such a strategy is cost/effective. Indeed, it is worthy of confirmation in larger population and for different degree on inter observer agreement as for LDCT findings of interest. Thus, only the definitive production of predictive models and the formal computation of their diagnostic accuracy will allow define the true value of these preliminary, yet intriguing, observations.

## Supplementary Information

**Additional file 1.** It provides a detailed description of LDCT scan findings (**e-Table 1**), and their association with CD (**e-Table 3**), RD (**e-Table 4**) and OD (**e-Table 5**) in the whole sample of 974 screened patients (**e-Table 2**).** e-Table 6** reports the radiologists’ agreement per LDCT finding.

## Data Availability

Data related to the findings reported in our manuscript are available from the corresponding author to all interested researchers upon reasonable request and with the permission of the Regional Department, because of stringent legal restrictions regarding the privacy policy on personal in-formation in Italy (national legislative decree on privacy policy n. 196/30 June 2003). For these reasons our dataset cannot be made available on public data deposition.

## Abbreviations

LDCT scan: Low-dose CT scan
CD: Cardiovascular diseases
RD: Respiratory diseases
OD: Oncological diseases
