# Supplementary material for: The additional value of lung cancer screening program in identifying unrecognized diseases
Source: BMC Pulm Med. 2022 Jan 31;22:48. doi: 10.1186/s12890-022-01826-1 (PMC8802423; doi:10.1186/s12890-022-01826-1)
Supplement: Supplementary file 1 — Additional file 1. It provides a detailed description of LDCT scan findings (e-Table 1), and their association with CD (e-Table 3), RD (e-Table 4) and OD (e-Table 5) in the whole sample of 974 screened patients (e-Table 2). e-Table 6 reports the radiologists’ agreement per LDCT finding. [file 12890_2022_1826_MOESM1_ESM.docx]

**The additional value of lung cancer screening program in identifying unrecognized diseases**

Panaiotis Finamore^1^, Luigi Tanese^2^, Filippo Longo^3^, Domenico De Stefano^2^, Claudio Pedone^1^, Laura Angelici^4^, Nera Agabiti^4^, Silvia Cascini^4^, Marina Davoli^4^, Bruno Beomonte Zobel^2^, Raffaele Antonelli Incalzi^1^, Pierfilippo Crucitti^3^

^1^ Campus Bio-Medico di Roma University, Unit of Geriatrics, Department of Medicine and Surgery, Rome, Italy.

^2^ Campus Bio-Medico di Roma University, Unit of Imaging Center, Department of Medicine and Surgery, Rome, Italy.

^3^ Campus Bio-Medico di Roma University, Unit of Thoracic Surgery, Department of Medicine and Surgery, Rome, Italy.

^4^ Dipartimento di epidemiologia del Servizio sanitario regionale, Regione Lazio, ASL Roma 1, Rome, Italy.

**Running head**: CT-scan screening program: beyond lung cancer

**Corresponding author**:

Filippo Longo, MD

Unit of Thoracic Surgery Campus Bio-Medico University

Via Alvaro del Portillo, 200

00128 Rome, Italy

Phone +39 06 22 541 8144

Fax +39 06 22 541 456

E-mail: [f.longo@unicampus.it](mailto:f.longo@unicampus.it)

**e-Table 1** Description of the LDCT scan abnormal findings

| ***Respiratory findings:*** |  |
| --- | --- |
| Centrilobular emphysema | Centrilobular areas of decreased attenuation, usually without visible walls, of nonuniform distribution |
| Paraseptal emphysema | Low-attenuating areas located adjacent to the pleura and septal lines with a peripheral distribution within the secondary pulmonary lobules. |
| Ground glass opacity | Haziness areas with preserved vessels and airways |
| Atelectasis areas | Areas of reduced volume, accompanied by increased attenuation (CT scan) in the affected part of the lung |
| Bronchiectasis | Dilated and thickened bronchi, with an increased bronchial/pulmonary artery diameter ratio |
| Diffuse fibrosis | Reticular opacities and honeycombing, with a predominantly peripheral and basal distribution |
| Subpleural fibrosis | Focal reticular or ground glass opacities with interlobular septal thickening in the supleural space |
| Paravertebral fibrosis | Focal reticular or ground glass opacities with interlobular septal thickening in the paravertebal space |
| Mucus plug | Hypodense soft-tissue density material in the bronchial lumen |
| Tracheal dilatation | Lumen diameter ≥ 30 mm |
| Focal pleural thickening | Thickening involving either the parietal or visceral pleura ≥ 3 mm |
| ***Cardiovascular findings:*** |  |
| Vascular calcifications | Calcifical wall-thickening of the aorta and the epi-aortic vessels |
| Coronary calcifications | Calcifical wall-thickening of a bronchus |
| Valvular calcification | Calcifical thickness of mitralic, aortic and tricuspidalic leaflets |
|  |  |
| ***Other findings:*** |  |
| Breast nodule | ≥ 5 mm |

**e-Table 2** Participants’ characteristics and LDCT scan findings (n=974).

| **Variables** | **N=974** |
| --- | --- |
| **Age (year)** | 62.2 (5.7) |
| Age (years ) |  |
| 52-56 | 293 (30.1%) |
| 59-64 | 365 (37.5%) |
| 65-78 | 315 (32.4%) |
| **Sex (M)** | 574 (58.9) |
| **Current smokers** | 678 (69.6) |
| **Former smokers** | 2.83 (29.1) |
| **Pack year** |  |
| **Professional exposure** | 22 (2.3) |
| **Chronic obstructive pulmonary disease** | 206 (21.7) |
| **Comorbidities** |  |
| Bronchial asthma | 16 (1.6) |
| Diabetes mellitus | 67 (6.9) |
| Hypertension | 335 (34.4) |
| Gastroesophageal reflux disease | 44 (4.5) |
| Hypercholesterolemia | 169 (17.4) |
| Hyperthyroidism | 1 (0.1) |
| Benign prostatic hyperplasia (BPH) | 58 (6) |
| **Lung nodules** |  |
| Nodules <=4 mm | 238 (24.4) |
| Nodules 5-6 mm | 175 (18) |
| Nodules 7-8 mm | 57 (5.9) |
| Nodules >=8 mm | 117 (12) |
| **LDCT findings** |  |
| Ground glass opacity | 59 (6.1) |
| Centrilobular emphysema | 289 (29.7) |
| Paraseptal emphysema | 97 (10) |
| Atelectasis areas | 91 (9.3) |
| Bronchial wall thickening | 389 (39.9) |
| Bronchiectasis | 82 (8.4) |
| Diffuse fibrosis | 15 (1.5) |
| Subpleural fibrosis | 358 (36.8) |
| Paravertebral fibrosis | 30 (3.1) |
| Mucus plug | 24 (2.5) |
| Tracheal deviation | 23 (2.4) |
| Focal pleural thickening | 84 (8.6) |
| Pleural effusion | 1 (0.1) |
| Vascular calcifications | 150 (46.2) |
| Coronary Calcifications | 335 (34.4) |
| Valve Calcifications | 152 (15.6) |
| Breast nodule | 17 (1.8) |

**e-Table 3** Association between participants’ characteristics and LDCT scan findings with cardiovascular diseases (n=84).

|  |  | **Univariable logistic regression** | | | **Multivariable logistic regression** | | |
| --- | --- | --- | --- | --- | --- | --- | --- |
|  | **N=84** | **OR** | **95%CI** | **P** | **OR** | **95%CI** | **P** |
| **Age** |  |  |  |  |  |  |  |
| 52-58 years | 14 (16.7) | Ref |  |  |  |  |  |
| 59-64 years | 36 (42.8) | 2.18 | 1.15-4.12 | 0.02 | - | - | - |
| 65-78 years | 34 (40.5) | 2.41 | 1.27-4.59 | <0.01 | - | - | - |
| **Sesso (M)** | 59 (70.2) | 1.72 | 1.06-2.79 | 0.03 | - | - | - |
| **Smoking** |  |  |  |  |  |  |  |
| Never smoker | 2 (2.4) | Ref |  |  |  |  |  |
| Former smoker | 18 (21.4) | 0.44 | 0.09-2.12 | 0.17 | - | - | - |
| Current smoker | 64 (76.2) | 0.68 | 0.15-3.07 | 0.97 | - | - | - |
| Pack year | 42.2 (22) | 1.01 | 1.00-1.02 | 0.06 | - | - | - |
| **Lung nodules** |  |  |  |  |  |  |  |
| No nodules | 31 (37) | Ref |  |  | Ref |  |  |
| Nodules <=8 mm | 45 (53.5) | 1.17 | 0.72-1.93 | 0.53 | 1.15 | 0.70-1.90 | 0.59 |
| Nodules >8 mm | 8 (9.5) | 1.10 | 0.58-2.09 | 0.78 | 0.95 | 0.50-1.83 | 0.43 |
| **Ground glass opacity** | 7 (8.3) | 1.46 | 0.64-3.34 | 0.36 | 1.51 | 0.66-3.47 | 0.33 |
| **Centrilobular emphysema** | 28 (33.3) | 1.21 | 0.75-1.94 | 0.44 | 1.09 | 0.67-1.77 | 0.72 |
| **Paraseptal emphysema** | 12 (14.3) | 0.58 | 0.82-3.03 | 0.17 | 1.34 | 0.69-2.60 | 0.38 |
| **Atelectasis areas** | 9 (10.7) | 1.18 | 0.57-2.45 | 0.65 | 1.13 | 0.54-2.35 | 0.75 |
| **Bronchial wall thickening** | 39 (46.4) | 1.34 | 0.85-2.10 | 0.21 | 1.25 | 0.80-1.98 | 0.33 |
| **Bronchiectasis** | 13 (15.5) | 2.18 | 1.15-4.13 | 0.02 | 2.07 | 1.07-3.97 | 0.03 |
| **Diffuse fibrosis** | 3 (3.6) | 2.71 | 0.75-9.80 | 0.13 | 2.14 | 0.58-7.93 | 0.26 |
| **Subpleural fibrosis** | 23 (27.4) | 0.63 | 0.38-1.03 | 0.06 | 0.60 | 0.36-0.99 | 0.05 |
| **Paravertebral fibrosis** | 4 (4.8) | 1.66 | 0.57-4.88 | 0.35 | 1.48 | 0.50-4.41 | 0.48 |
| **Mucus plugs** | 6 (7.1) | 3.72 | 1.43-9.65 | <0.01 | 3.65 | 1.38-9.64 | <0.01 |
| **Tracheal deviation** | 2 (2.4) | 1.04 | 0.24-4.56 | 0.95 | 0.76 | 0.17-3.41 | 0.72 |
| **Coronary calcifications** | 39 (46.4) | 1.74 | 1.11-2.73 | 0.02 | 1.40 | 0.88-2.24 | 0.16 |
| **Valve calcifications** | 23 (27.4) | 2.22 | 1.33-3.72 | <0.01 | 1.93 | 1.14-3.27 | 0.01 |
| **Breast nodule** | 2 (2.4) | 1.43 | 0.32-6.33 | 0.64 | 1.90 | 0.41-8.84 | 0.41 |

Categorical variables are expressed as frequency (%), while continuous variables as mean (SD). Adjustment variables in the multivariable logistic regression were age, sex and pack year.

**e-Table 4** Association between participants’ characteristics and LDCT scan findings with respiratory diseases (n=42).

|  |  | **Univariable logistic regression** | | | **Multivariable logistic regression** | | |
| --- | --- | --- | --- | --- | --- | --- | --- |
|  | **N=42** | **OR** | **95%CI** | **P** | **OR** | **95%CI** | **P** |
| **Age** |  |  |  |  |  |  |  |
| 52-58 years | 9 (21.4) | Ref |  |  |  |  |  |
| 59-64 years | 15 (35.7) | 1.35 | 0.58-3.14 | 0.95 | - | - | - |
| 65-78 years | 18 (42.9) | 1.91 | 0.85-4.33 | 0.12 | - | - | - |
| **Sesso (M)** | 27 (64.3) | 1.27 | 0.66-2.41 | 0.47 | - | - | - |
| **Smoking** |  |  |  |  |  |  |  |
| Former smoker | 11 (26.2) | - | - | - | - | - | - |
| Current smoker | 31 (73.8) | - | - | - | - | - | - |
| Pack year | 48.1 (25) | 1.01 | 0.99-1.02 | 0.10 | - | - | - |
| **Lung nodules** |  |  |  |  |  |  |  |
| No nodules | 6 (14.3) | Ref |  |  | Ref |  |  |
| Nodules <=8 mm | 20 (47.6) | 0.88 | 0.44-1.76 | 0.71 | 0.85 | 0.42-1.72 | 0.65 |
| Nodules >8 mm | 16 (38.1) | 1.96 | 0.84-4.56 | 0.12 | 1.71 | 0.73-4.03 | 0.22 |
| **Ground glass opacity** | 3 (7.1) | 1.20 | 0.36-4.01 | 0.76 | 1.19 | 0.36-4.00 | 0.77 |
| **Centrilobular emphysema** | 20 (47.6) | 2.24 | 1.20-4.17 | 0.01 | 2.12 | 1.13-3.97 | 0.02 |
| **Paraseptal emphysema** | 10 (23.8) | 3.03 | 1.44-6.38 | <0.01 | 2.69 | 1.26-5.74 | 0.01 |
| **Atelectasis areas** | 5 (11.9) | 1.33 | 0.51-3.47 | 0.56 | 1.27 | 0.48-3.35 | 0.62 |
| **Bronchial wall thickening** | 19 (45.2) | 1.26 | 0.67-2.34 | 0.47 | 1.17 | 0.62-2.18 | 0.63 |
| **Bronchiectasis** | 4 (9.5) | 1.15 | 0.40-3.31 | 0.79 | 1.06 | 0.36-3.06 | 0.92 |
| **Diffuse fibrosis** | 2 (4.8) | 3.53 | 0.77-16.2 | 0.10 | 2.81 | 0.60-13.25 | 0.19 |
| **Subpleural fibrosis** | 16 (38.1) | 1.06 | 0.56-2.01 | 0.85 | 1.02 | 0.54-1.93 | 0.96 |
| **Paravertebral fibrosis** | 1 (2.4) | 0.76 | 0.10-5.71 | 0.79 | 0.65 | 0.09-4.97 | 0.68 |
| **Mucus plugs** | 2 (4.8) | 2.07 | 0.47-9.09 | 0.34 | 2.03 | 0.46-9.06 | 0.35 |
| **Tracheal deviation** | 1 (2.4) | 1.19 | 0.15-9.15 | 0.87 | 0.95 | 0.12-7.44 | 0.96 |
| **Coronary calcifications** | 18 (42.9) | 1.46 | 0.78-2.72 | 0.24 | 1.24 | 0.64-2.38 | 0.53 |
| **Valve calcifications** | 8 (19) | 1.28 | 0.58-2.84 | 0.53 | 1.12 | 0.50-2.49 | 0.79 |
| **Breast nodule** | 1 (2.4) | 1.40 | 0.18-10.79 | 0.75 | 1.57 | 0.20-12.7 | 0.67 |

Categorical variables are expressed as frequency (%), while continuous variables as mean (SD). Adjustment variables in the multivariable logistic regression were age, sex and pack year.

**e-Table 5** Association between participants’ characteristics and LDCT scan findings with oncologic diseases (n=48).

|  |  | **Univariable logistic regression** | | | **Multivariable logistic regression** | | |
| --- | --- | --- | --- | --- | --- | --- | --- |
|  | **N=48** | **OR** | **95%CI** | **P** | **OR** | **95%CI** | **P** |
| **Age** |  |  |  |  |  |  |  |
| 52-58 years | 8 (16.7) | Ref |  |  |  |  |  |
| 59-64 years | 16 (33.3) | 1.63 | 0.69-3.87 | 0.26 | - | - | - |
| 65-78 years | 24 (50) | 2.94 | 1.30-6.65 | 0.01 | - | - | - |
| **Sesso (M)** | 21 (43.8) | 0.66 | 0.26-1.69 | 0.38 | - | - | - |
| **Smoking** |  |  |  |  |  |  |  |
| Never smoker | 1 (2.1) | Ref |  |  |  |  |  |
| Former smoker | 10 (20.8) | 0.10 | 0.01-1.15 | 0.11 | - | - | - |
| Current smoker | 37 (77.1) | 0.11 | 0.01-1.17 | 0.13 | - | - | - |
| Pack year | 42.4 (22) | 1.01 | 0.99-1.03 | 0.13 | - | - | - |
| **Lung nodules** |  |  |  |  |  |  |  |
| No nodules | 15 (31.3) | Ref |  |  | Ref |  |  |
| Nodules <=8 mm | 14 (29.1) | 0.77 | 0.37-1.61 | 0.48 | 0.67 | 0.32-1.43 | 0.30 |
| Nodules >8 mm | 19 (39.6) | 4.88 | 2.39-9.96 | <0.01 | 4.45 | 2.19-9.45 | <0.01 |
| **Ground glass opacity** | 5 (10.4) | 1.60 | 0.35-7.25 | <0.01 | 1.73 | 0.65-4.62 | 0.27 |
| **Centrilobular emphysema** | 19 (39.6) | 1.35 | 0.52-3.56 | 0.54 | 1.59 | 0.87-2.92 | 0.14 |
| **Paraseptal emphysema** | 7 (14.6) | 1.48 | 0.41-5.26 | 0.55 | 1.53 | 0.65-3.58 | 0.33 |
| **Atelectasis areas** | 9 (18.8) | 4.02 | 1.36-11.82 | 0.01 | 2.26 | 1.04-4.92 | 0.04 |
| **Bronchial wall thickening** | 22 (45.8) | 1.56 | 0.55-4.46 | 0.40 | 1.15 | 0.63-2.07 | 0.66 |
| **Bronchiectasis** | 5 (10.4) | 1.18 | 0.26-5.29 | 0.83 | 1.05 | 0.40-2.77 | 0.92 |
| **Diffuse fibrosis** | 1 (2.1) | 1.39 | 0.18-10.77 | 0.75 | 1.22 | 0.16-9.69 | 0.85 |
| **Subpleural fibrosis** | 18 (37.5) | 0.84 | 0.30-2.41 | 0.75 | 0.95 | 0.52-1.74 | 0.86 |
| **Paravertebral fibrosis** | 3 (6.3) | 3.54 | 0.75-16.71 | 0.11 | 0.93 | 0.55-6.71 | 0.30 |
| **Mucus plugs** | 3 (6.3) | 4.75 | 0.98-23.01 | 0.05 | 2.77 | 0.77-9.98 | 0.12 |
| **Tracheal deviation** | 4 (8.3) | 6.77 | 2.03-22.51 | <0.01 | 6.95 | 2.04-23.69 | <0.01 |
| **Coronary calcifications** | 22 (45.8) | 2.89 | 1.10-7.61 | 0.03 | 1.62 | 0.87-3.01 | 0.13 |
| **Valve calcifications** | 10 (20.8) | 0.84 | 0.24-2.97 | 0.79 | 1.22 | 0.58-2.55 | 0.60 |
| **Breast nodule** | 2 (4.2) | 2.64 | 0.59-11.89 | 0.21 | 1.88 | 0.40-8.87 | 0.43 |

Categorical variables are expressed as frequency (%), while continuous variables as mean (SD). Adjustment variables in the multivariable logistic regression were age, sex and pack year. The following LDCT scan findings were not represented in this cohort: pulmonary trunk ectasia, pericardium pouring and thoracic cage abnormalities.

**e-Table 6** Radiologists’ agreement per finding on a sample of 50 LDCT scans.

| **LDCT scan finding** | **Adjudicator 1** | **Adjudicator 2** | **Cohen’s k (95%CI)** | **p-value** |
| --- | --- | --- | --- | --- |
| Ground glass opacity | 5 (10) | 5 (10) | 0.55 (0.17-0.94) | <0.01 |
| Centrilobular emphysema | 13 (26) | 13 (26) | 0.85 (0.68-1.01) | <0.01 |
| Paraseptal emphysema | 8 (16) | 7 (14) | 0.92 (0.77-1.07) | <0.01 |
| Atelectasis areas | 3 (6) | 4 (8) | 0.54 (0.08-0.10) | <0.01 |
| Bronchial wall thickening | 32 (64) | 31 (62) | 0.44 (0.19-0.70) | <0.01 |
| Bronchiectasis | 12 (24) | 10 (20) | 0.65 (0.40-0.91) | <0.01 |
| Subpleural fibrosis | 3 (6) | 3 (6) | 1 | <0.01 |
| Mucus plugs | 6 (12) | 1 (2) | 0.26 (0.15-0.67) | <0.01 |
| Tracheal deviation | 1 (2) | 1 (2) | 1 | <0.01 |
| Focal pleural thickening | 1 (2) | 1 (2) | 1 | <0.01 |
| Coronary Calcifications | 15 (30) | 13 (26) | 0.90 (0.77-1.03) | <0.01 |
| Valve Calcifications | 6 (12) | 5 (10) | 0.90 (0.64-1.11) | <0.01 |
| Breast nodule | 3 (6) | 3 (6) | 0.65 (0.18-1.01) | <0.01 |

Data are expressed as frequency (%).
